# Deodorization of Iodoform

**Published:** 1896-04

**Authors:** 


### Deodorization of Iodoform.

The *Journal de Medecine* of January 19th, republishes the following from the *Presse Medicale* prefacing it with the query: Why not use di-iodoform? This has all the properties of simple iodoform without any odor, as Hallopeau demonstrated before the Societe de Therapie, in 1894. Jaksch observed that all the antiseptics with a specific odor possessed the property of disguising the odor of iodoform, while their own became scarcely perceptible. Thymol, naphthalin, tar and creolin can be used in this way, preferably the latter, in the proportion of 1 to 2 percent. This produces a brownish powder with a mild aromatic odor. Oppler suggests pulverized coffee in a proportion of $\frac{1}{3}$ to $\frac{1}{4}$. Ten percent conmarin or vanillin disguises the odor of iodoform, and also acid of cinnamon in equal quantity to the iodoform. Laura Goodman observed that after a menthol pencil had been left an hour or two in a bottle of iodoform, the odor had disappeared. There is a bituminous iodoform made by a secret process; and brownish mica-like scales, with a metallic luster, are produced by blending tar with the iodoform to the point of deodorization. Kay made a 10 percent paste of tar, that had no odor except that of the tar; 5 percent produces a powder in which the iodoform is not recognized. Negel's pills are made thus: 3 grams iodoform, 15 grams tar and 0.60 grams extract of opium for 120 pills administered eight a day. Cantrelli mixes equal parts of iodoform, essence of menthol and essence of lavender.

